# Transcriptomic study reveals lncRNA-mediated downregulation of innate immune and inflammatory response in the SARS-CoV-2 vaccination breakthrough infections

**DOI:** 10.3389/fimmu.2022.1035111

**Published:** 2022-11-18

**Authors:** Partha Chattopadhyay, Pallavi Mishra, Priyanka Mehta, Jyoti Soni, Rohit Gupta, Bansidhar Tarai, Sandeep Budhiraja, Rajesh Pandey

**Affiliations:** ^1^ Division of Immunology and Infectious Disease Biology, INtegrative GENomics of HOst-PathogEn (INGEN-HOPE) laboratory, CSIR-Institute of Genomics and Integrative Biology (CSIR-IGIB), Delhi, India; ^2^ Academy of Scientific and Innovative Research (AcSIR), Ghaziabad, India; ^3^ Max Super Speciality Hospital (A Unit of Devki Devi Foundation), Max Healthcare, Delhi, India

**Keywords:** adaptive immunity, innate immunity, vaccination breakthrough, SARS-CoV-2, repeat elements, lncRNAs, topologically associated domains (TADs)

## Abstract

**Introduction:**

The emergence of multiple variants of concerns (VOCs) with higher number of Spike mutations have led to enhanced immune escape by the SARS-CoV-2. With the increasing number of vaccination breakthrough (VBT) infections, it is important to understand the possible reason/s of the breakthrough infections.

**Methods:**

We performed transcriptome sequencing of 57 VBT and unvaccinated COVID-19 patients, followed by differential expression and co-expression analysis of the lncRNAs and the mRNAs. The regulatory mechanism was highlighted by analysis towards repeat element distribution within the co-expressed lncRNAs, followed by repeats driven homologous interaction between the lncRNAs and the promoter regions of genes from the same topologically associated domains (TAD).

**Results:**

We identified 727 differentially expressed lncRNAs (153 upregulated and 574 downregulated) and 338 mRNAs (34 up- and 334 downregulated) in the VBT patients. This includes *LUCAT1*, *MALAT1*, *ROR1-AS1*, *UGDH-AS1* and *LINC00273* mediated modulation of immune response, whereas *MALAT1*, *NEAT1* and *GAS5* regulated inflammatory response in the VBT. LncRNA-mRNA co-expression analysis highlighted 34 lncRNAs interacting with 267 mRNAs. We also observed a higher abundance of Alu, LINE1 and LTRs within the interacting lncRNAs of the VBT patients. These interacting lncRNAs have higher interaction with the promoter region of the genes from the same TAD, compared to the non-interacting lncRNAs with the enrichment of Alu and LINE1 in the gene promoter.

**Discussion:**

Significant downregulation and GSEA of the TAD gene suggest Alu and LINE1 driven homologous interaction between the lncRNAs and the TAD genes as a possible mechanism of lncRNA-mediated suppression of innate immune/inflammatory responses and activation of adaptive immune response. The lncRNA-mediated suppression of innate immune/inflammatory responses and activation of adaptive immune response might explain the SARS-CoV-2 breakthrough infections with milder symptoms in the VBT. Besides, the study also highlights repeat element mediated regulation of genes in 3D as another possible way of lncRNA-mediated immune-regulation modulating vaccination breakthroughs milder disease phenotype and shorter hospital stay.

## Introduction

On November 26, 2021, WHO declared yet another variant of concern (VOC) B.1.1.529, with an unusually high number of mutations (32 mutations) in the spike region, the main antigenic target of vaccine-elicited antibodies (1). Despite the rapid vaccination program globally, the Omicron variant had very high transmission rate and low susceptibility to both vaccine-elicited neutralizing antibodies as well as therapeutic monoclonal antibodies (2). This underscored the global importance and effort towards understanding the efficacy of the existing vaccines/monoclonal antibodies. Many studies have been conducted to investigate the efficacy of existing vaccines against the emerging variants, and found a lower vaccination efficiency against the Omicron variants (3–5). This has spurred effort towards tailoring the available vaccine for Omicron specificity. However, another aspect of vaccination breakthrough (VBT) infection remains unsolved: despite receiving the immunizations that were meant to protect them/us from infection, what enables the virus to infect remains elusive. Few possible explanations could be the mutations in the spike protein of the virus, which helps in differential immune escape, lower titer of neutralizing antibody, and less sensitivity towards the vaccine-elicited neutralizing antibody, however these triggers another confutation: if the neutralizing antibody was not sufficient enough to counter the infection, why the conventional innate immunity was not triggered?

The key to the above question possibly lies in the fact that a vaccinated COVID-19 positive individual has milder symptoms compared to an unvaccinated COVID-19 patient (6, 7). This suggests a differential host response in the vaccination breakthrough infection. Long noncoding RNAs (lncRNAs), as a key component of the human transcriptome, and because of their gene regulatory role, are crucial factors to explore/elucidate in order to understand the variable host response in the vaccine breakthrough infection. Furthermore, prior research by our lab and others has emphasized the function of lncRNAs in the SARS-CoV-2 infection and how it influences differential disease severities (8–10). Therefore, we conducted this first-of-its-kind study to understand the role of lncRNAs in the vaccination breakthrough infection, which might explain why despite the vaccination, the individuals were infected with the SARS-CoV-2.

In this study, we performed transcriptome sequencing of 57 vaccination breakthroughs and unvaccinated SARS-CoV-2 infected individuals. Through a combination of differential expression analysis of lncRNAs, mRNAs, and co-expression analysis of the lncRNAs-mRNAs, we observed a decreased inflammatory and innate immune response, but increased adaptive immune response in the vaccination breakthrough infections. Multiple studies have highlighted higher abundance of repeat elements within the lncRNA sequences and the role of repeat elements in modulating lncRNA functions (11, 12). We have also shown that Alu and LINE1-driven homology-based interaction of lncRNAs with the promoter region of genes from same topologically associated domains (TAD) is one of the possible mechanisms of lncRNA-mediated modulation of innate immune response in the VBT infections. Together, the findings highlight the lncRNAs-mediated modulation of innate immune response, adaptive immune response and inflammatory response, which could possibly explain the VBT and the milder disease severity in these patients with shorter hospital stay.

## Materials and methods

### Patient cohort, sampling and data collection

The patients were admitted to a tertiary care center (Max Super Speciality Hospital, Delhi, India) with confirmed COVID-19 positive status based on qRT-PCR result during February-April 2021. A subset of the COVID-19 positive individuals with very mild symptoms were kept in home isolation under medical observation. Both nasopharyngeal and oropharyngeal swabs were collected in VTM by paramedical staff on the day of hospital admission or sampling during home isolation. Viral RNA from VTM was isolated using QIAmp viral mini kit (Qiagen, Cat. No. 52906) and SARS-CoV-2 detection and quantification was performed using TRUPCR SARS-CoV-2 kit (3B BlackBio Biotech India Ltd., Cat. No. 3B304), with a cycle threshold of 35. Sequencing of SARS-CoV-2 genome was performed using Illumina COVIDSeq Test (Cat. No 20043675) as per manufacturer’s reference guide (#1000000126053v04) for all the COVID-19 positive individuals to confirm the viral infection. The demographic and clinical details along with COVID-19 vaccination history of the patients were collected from the electronic health record as per standard practices. The individuals were segregated into two groups based on their COVID-19 vaccination status: *Vaccination breakthrough* - individuals infected with SARS-CoV-2 after vaccination, and *Unvaccinated* - individuals infected with SARS-CoV-2 prior to COVID-19 vaccination. All study procedures were in accordance with the Declaration of Helsinki and approved by the Institutional Ethics Committee of the CSIR-Institute of Genomics and Integrative Biology (CSIR-IGIB) and the MAX Super Specialty Hospital. Informed consent was obtained from all individuals or their legal guardians.

### Library preparation and sequencing

RNA sequencing libraries were prepared using Illumina TruSeq^®^ Stranded Total RNA Library Prep Gold (cat. no 20020599) from a total of 250 ng RNA isolated from the naso/oropharyngeal swabs of COVID-19 positive individuals, as per manufacturer’s reference guide (1000000040499 v00) and our previous study (13). Cytoplasmic and mitochondrial rRNA were removed using Ribo-Zero rRNA removal beads. The purified RNA was fragmented to achieve size uniformity using divalent cation under an elevated temperature. The cDNA was synthesized from the fragmented RNA using SuperScript IV reverse transcriptase, followed by RNA strand digestion from the RNA-DNA hybrid using RNASE-H, and synthesis of the second strand of cDNA. The 3’ blunt ends of the double stranded cDNA were adenylated prior to addition of indices and amplification. The final library was purified using AMPure XP (Beckman Coulter, A63881), followed by quality check using Agilent 2100 bioanalyzer and High Sensitivity DNA Kit. The libraries were sequenced on NextSeq 2000 platform using P2 reagent kit, 2 x 151 cycles and at a final loading concentration of 650 pM.

### Quality control, mapping to reference and identification of DE-lncRNAs/genes

FastQC was used to determine the quality of the raw reads, followed by trimming of adapter sequences using Trimmomatic (14). Reads are mapped to the human reference transcriptome (Genome Reference Consortium Human Build 38, release 107, corresponding to the GenBank Assembly ID GCA_000001405.28) using Salmon quasi mapping tool to quantify read abundance or transcript expression levels (15). The lncRNA expression profile was quantified using LNCipedia transcript annotation v5.2 as reference, with 200 bp size cut-off (16). The protein coding gene expression was quantified using Ensembl protein coding gene annotation as reference. To identify significantly differentially expressed lncRNAs and the protein coding genes, DESeq2 was applied with Wald’s test as statistical algorithm, with a cut-off of p-adjusted value of ≤ 0.05, and Log2 fold change of ≥ ± 1.5 (17). The Log fold change was plotted against p-adjusted value using VolcanoseR R package (18).

### LncRNA-mRNA co-expression analysis

Pearson correlation analysis between the lncRNAs and the mRNAs expression was performed to identify the lncRNA-mRNA co-expression. A Pearson correlation coefficient (PCC) value of ≥ ± 0.9 (at p value ≤ 0.05), and at least one correlation between the differentially expressed lncRNA and mRNA expression was considered to be significant. The correlation coefficient between lncRNA-mRNA was used to filter the candidates for the interaction network. The candidate lncRNAs and mRNAs were scanned against NPInter v4.0 and LncRRIsearch v1.0 to build the interaction network using MCODE and Cytoscape (19–22). A betweenness filter of 0.9 was applied to simplify the complex interaction network. The tools are house to experimentally validated lncRNAs and mRNAs, indicative of their plausible functional role.

### Gene set enrichment analysis of network interacting mRNAs

The mRNAs interacting with the lncRNAs were used to perform the GSEA. Fast GSEA (fGSEA) R package was used and GSEA was performed against the KEGG database (23, 24). A cut-off of p-adj ≤ 0.05 was applied to select the significant pathways. The Normalized Enrichment Score (NES) and the Log2 fold change of the genes involved in the pathways were visualized using ComplexHeatmap R package.

### Pathway enrichment analysis of the interacting LncRNAs

The lncRNAs interacting with the mRNAs were used to perform Pathway enrichment analysis. ncPath web server was used to perform the pathway enrichment against KEGG database, and a cut-off of p-adj ≤ 0.05 was applied to select the significant pathways and was visualized using ggplot2 R package (25).

### Repeat element distribution analysis

The network interacting lncRNAs were selected for repeat element distribution analysis using RepeatMasker web server (26). The analysis was performed using rmblast algorithm and Dfam 3.0 database (27). The repeat elements were segregated with respect to class, sub-class, lengths of lncRNA covered, and presence on sense/antisense strand, and were compared with the repeat elements present within the lncRNAs with no network interaction but significant PCC. Statistical analysis was performed using GraphPad Prism 9.2.

### Topologically associated domain gene analysis

The protein coding genes present within the same TAD as of the interacting lncRNAs were fetched from the TADKB database (28). Experimental TADs from lung fibroblast cell line, IMR90, were considered as it is the closest to our sample type, amongst the other available cell lines with the TAD information. GSEA was performed using fGSEA on the protein coding genes retrieved from the TADs. LncRNA-Gene triplex formation at the promoter region of the genes was predicted using Triplex Domain Finder from the Regulatory Genomics Toolbox (29). The total number of triplex formations by the interacting lncRNAs was compared against the non-interacting lncRNAs. The abundance of repeat elements within the interacting region of the lncRNAs, and the promoter region of the genes were performed using RepeatMasker as stated earlier.

## Results

### Patient cohort characterization: classification and clinical evaluation

**Figure 1:**
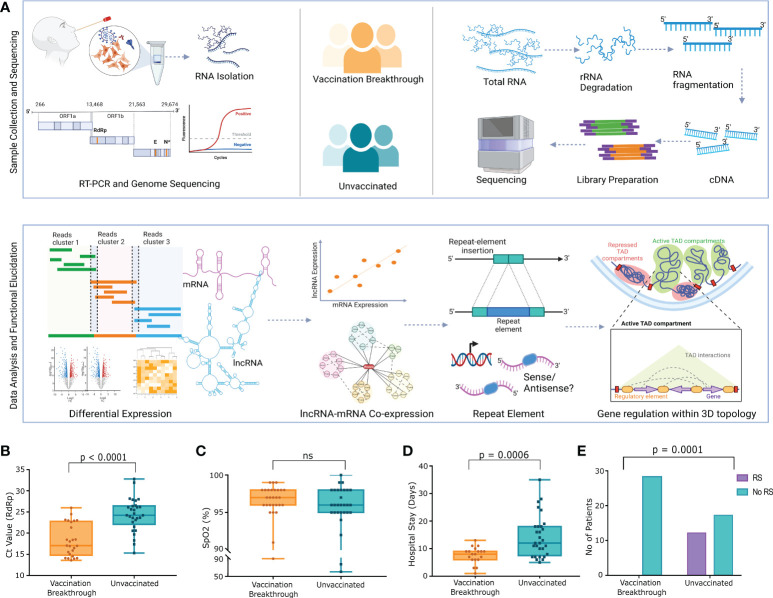
Study design, experimental workflow, and clinical data of the SARS-CoV-2 Infected Individuals across VBT and unvaccinated. **(A)** Study design and experimental workflow including the sample collection, SARS-CoV-2 genome sequencing, human host transcriptome, data analysis and the functional interpretation. **(B)** Ct value of the SARS-CoV-2 RdRp gene, **(C)** SpO_2_ (%) levels of the VBT and unvaccinated individuals, **(D)** Duration of hospital stay, and **(E)** Number of patients requiring respiratory support between the VBT and the unvaccinated individuals. Upper bar shows statistical significance.

We recruited 57 COVID-19 patients to understand the role of lncRNAs in the vaccination breakthrough infections. The patients were stratified into two groups based on their vaccination status; *Vaccination Breakthrough* (n=28) with ChAdOx1 nCoV-19 vaccination prior to infection and *Unvaccinated* (n=29) including patients without any prior vaccination. Briefly, after qRT-PCR and sequence-based confirmation of COVID-19 status, we also sequenced the whole transcriptome of the 57 individuals, followed by transcript level differential expression analysis of the lncRNAs, mRNAs, lncRNA-mRNA co-expression analysis, repeat element distribution analysis and lncRNA-gene interaction analysis within the topologically associated domains (TAD). **Figure 1A** highlights the experimental design, sample collection, sequencing, data analysis and the inferences drawn.

The demographic and clinical data of the patients are summarized in the **Supplementary Table S1**. The median Ct value of the *RdRp* gene was significantly lower in the breakthrough infections, indicating a higher viral load in the VBT patients (**Figure 1B**). Interestingly, despite higher viral load, the median SpO_2_ was higher in the VBT (**Figure 1C**). The duration of hospital stay was also significantly lower in the VBT (**Figure 1D**), and none of the patients with the breakthrough infection required respiratory support, where few unvaccinated patients required respiratory support (**Figure 1E**). These, in summary, highlight that despite higher viral load in the VBT patients, the overall disease severity was lower compared to the unvaccinated individuals.

### LncRNA-mediated increased immune response and decreased inflammatory response in the vaccination breakthrough

**Figure 2:**
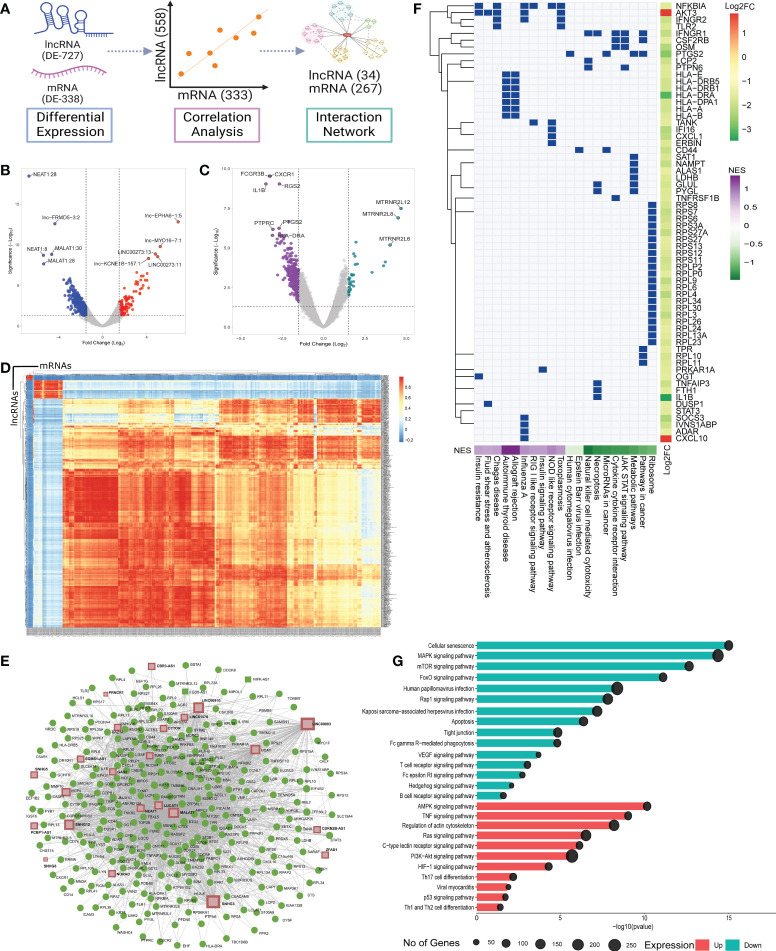
Differential expression, Co-expression and functional Role of the lncRNAs and the mRNAs between the vaccination breakthroughs and the unvaccinated. **(A)** Illustration of the DE analysis of the lncRNAs and the mRNAs, lncRNA-mRNA co-expression and interactions. **(B)** and **(C)** Volcano plot of the differential expression of the lncRNAs and the mRNAs between the vaccination breakthroughs and the unvaccinated, respectively. **(D)** Heatmap representing Pearson correlation coefficient between the DE lncRNAs and the mRNAs. **(E)** LncRNA-mRNA interaction network with Green dots representing the mRNAs and brown squares for the lncRNAs. **(F)** GSEA of the network interacting genes. Green box indicates involvement of a gene in a specific pathway. Side color bar indicates Log2 fold change of the genes, while the bottom color bar indicates the Normalized Enrichment Score (NES) of the pathways. **(G)** Pathway enrichment of the network interacting lncRNAs. Color represents the expression of the pathways, and the circle size highlights the number of genes involved in the pathways.

In order to understand the role of lncRNAs in modulating the disease trajectory between VBT and unvaccinated, we performed transcript level differential expression (DE) analysis of the lncRNAs. We identified a total of 727 differentially expressed (DE) lncRNAs (p-adj ≤ 0.05, Log2 fold change ≥ ± 1.5), with 153 upregulated and 574 downregulated in the VBT patients (**Figures 2A, B**, **Supplementary Table S2**). Out of the 727 DE-lncRNA transcripts, 32 lncRNA transcripts, corresponding to 15 lncRNA genes have known functions associated with the immune/inflammatory response. Further, out of the 12 lncRNA genes, 7 lncRNAs are reported to modulate immune/inflammatory responses in infectious diseases. Out of the 7 lncRNAs, *LUCAT1, MALAT1, NEAT1* and *GAS5* were downregulated in the VBT. *LUCAT1* is a negative regulator of interferon response, and downregulation of *LUCAT1* suggests an activated interferon response (30). *MALAT1* is known to negatively regulate the immune response and positively regulate the inflammatory response in the infected individuals, therefore downregulation of *MALAT1* in the breakthrough infection suggests an upregulated immune response and downregulated inflammatory response (31, 32). *NEAT1* and *GAS5* also act as pro-inflammatory lncRNAs, and their downregulation is an indicator of decreased inflammatory response in the VBT (33, 34). Amongst the upregulated lncRNAs, *ROR1-AS1, UGDH-AS1* and *LINC00273* were upregulated in the VBT. The upregulation of *ROR1-AS1* and *LINC00273* indicates a heightened immune response, whereas upregulation of *UGDH-AS1* suggests a decreased disease severity possibly modulated by *MOV10* and *EDN1* (8). In addition to these lncRNAs, functional interpretation of other DE lncRNAs are summarized in the **Table 1**.

**Table 1:** Functional role of the DE lncRNAs in the Immune and Inflammatory response.

lncRNAs	Expression	Normal Function	Outcome	Reference
*NIFK-AS1*	Upregulated	Negatively Regulates M2 polarization and Macrophage activation	Suppressed Innate Immunity	(35)
*NORAD*		Negatively regulates cytokine response through miR-485	Suppressed Inflammatory Response	(36, 37)
*SGMS1-AS1*		Negatively regulates cytokine response through miR-106a-5p		(38, 39)
*PRNCR1*		Positively regulates MAPK signaling by CCND2	Enhanced Adaptive Immunity	(40)
*FGD5-AS1*	Downregulated	Positively Regulates M2 polarization and Macrophage activation	Suppressed Innate Immunity	(41)
		Negatively regulates CD8 T cell activation through PD-L1 expression	Enhanced Adaptive Immunity	(42)
*LUCAT1*		Negative feedback regulator of IFN genes		(30)
*SNHG12*		Negatively regulates CD8 T cell activation through PD-L1 and IL6R		(43)
*SNHG16*		Negatively regulates CD8 T cell activation through CD73		(44)
*UCA1 *		Negatively regulates CD8 T cell activation through PD-L1		(45)
*MALAT1*		Negatively regulates IL10 mediated immune response		(31)
		Positively regulates TNF-α, IL6, IL1β	Suppressed Inflammatory Response	(10)
*NEAT1*		Positively regulates TNF-α, IL6, IL1β		(10)
*TUG1*		Positively regulates TNF-α, IL6, IL1β		(46)
*UCA1*		Positively regulates TNF-α, IL6, IL1β		(47)

### LncRNA-mRNA interaction modulates innate immune and inflammatory response

Most often, lncRNAs exert their function by modulating the protein coding genes (mRNAs). Therefore, to understand the possible lncRNA-mediated gene regulation, we performed DE analysis of the protein coding genes. We identified 338 DE genes (p-adj ≤ 0.05, Log2 fold change ≥ ± 1.5), 34 upregulated and 304 downregulated in the VBT compared to the unvaccinated (**Figures 2A, C**, **Supplementary Table S3**). We performed Pearson correlation analysis between the DE lncRNAs and the mRNAs. Significant Pearson correlation coefficient (PCC) was considered at R ≥ ± 0.9, p value ≤ 0.05 and at least one significant PCC was considered as co-expression of the lncRNA and the mRNA (**Figures 2A, D**). Out of the 727 DE-lncRNAs and 338 mRNAs, we found 558 lncRNAs and 331 mRNAs to be co-expressed (**Supplementary Table S4**). In order to identify genes co-expressed explicitly within the VBT and the unvaccinated groups, we have also performed the Pearson correlation analysis on the DE genes for individual groups. We then identify co-expressed genes unique to the VBT and the unvaccinated groups and performed Gene Ontology enrichment. While we observed immune response related GO terms to be enriched in the VBT-unvaccinated combined correlation analysis (**Supplementary Figure S1A**), metabolism/homeostasis related GO terms were enriched in the VBT (**Supplementary Figure S1B**) and adaptive immune response related GO terms were enriched in the unvaccinated group (**Supplementary Figure S1C**). Gene expression dynamics revealed that the metabolism/homeostasis related pathways were positively regulated in the VBT group, while the adaptive immune response related pathways were negatively regulated in the unvaccinated group.

We then used the lncRNA-mRNA co-expression to construct an interaction network. The network was constructed using MCODE, Cytoscape and a betweenness filter of 0.9 was applied to simplify the complex interaction network. LncRNAs with more than one transcript were merged into one while building the interaction network. We found 34 lncRNAs to be interacting with the 267 mRNAs (**Figures 2A, E**). To understand the possible lncRNA-mRNA interaction mediated perturbation of biological pathways, we performed gene set enrichment analysis (GSEA) on the network interacting genes (**Figure 2F**, **Supplementary Table S5**). We found inflammatory pathways like cytokine-cytokine receptor interaction, NK cell mediated cytotoxicity, necroptosis, JAK-STAT signaling pathways to be suppressed, while adaptive immune response and antiviral response related pathways like NLR and RLR signaling were activated in the VBT. Interestingly, ribosomal pathways were suppressed in the VBT, and multiple studies suggest hijacking of the ribosomal translation machinery by the viruses to facilitate viral replication. This could possibly explain the higher viral load in the VBT patients.

The pathway enrichment analysis of the interacting lncRNAs also highlights decreased inflammatory response (**Figure 2G**, **Supplementary Table S6**). Interestingly, some of the innate immune response associated pathways, such as AMPK signaling, PI3K-AKT signaling, and Ras signaling were also suppressed in the VBT. Together, these findings highlight a decreased innate immune and inflammatory response pathway but increased adaptive immune response pathway in the vaccination breakthrough patients.

### Repeat elements within LncRNAs as possible modulators of LncRNA-mediated immune response regulation

**Figure 3:**
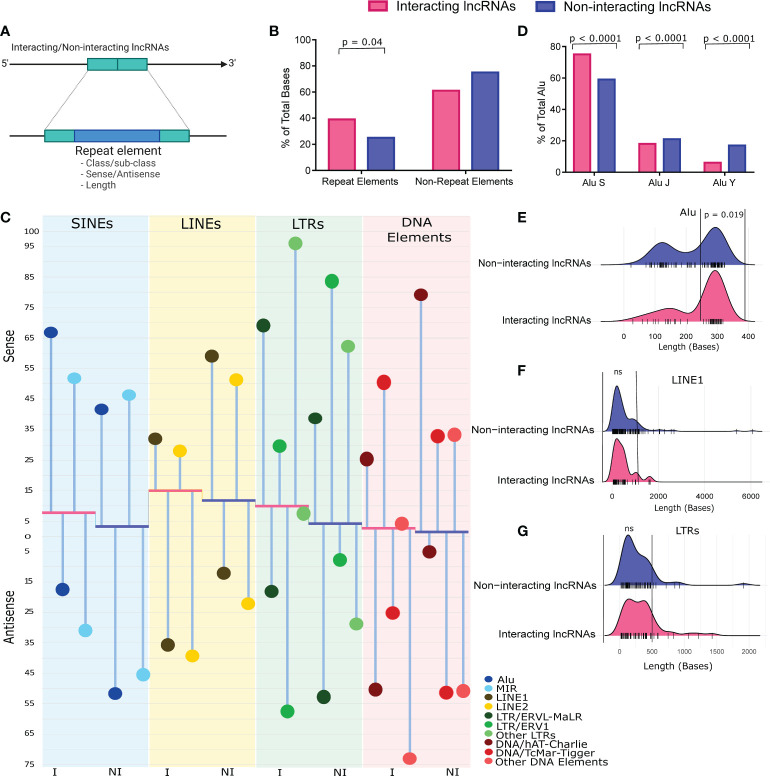
Repeat element abundance, class/subclass, strand-specific differential presence and size range of the Repeats. **(A)** Working model of the repeat element abundance analysis. **(B)** Repeat element abundance between the vaccination breakthroughs and the unvaccinated. Total bases covered by repeat elements were normalized against total lncRNA length. **(C)** Repeat element class/sub-class, and their strand specific distribution between the two groups. Abundance of major repeat classes are represented by bars, while height of bars from baseline indicates the abundance of repeats normalized to the lncRNA length. The red base of the plot corresponds to interacting lncRNAs **(I)**, while the blue base corresponds to the non-interacting lncRNAs (NI). Abundance of repeat sub-classes are represented as %age of the repeat class (lollipop plot). **(D)** Abundance of Alu subfamily between the two groups. Data is represented as %age of total Alu, normalized against total bases covered by the Alus. **(E–G)** Size distribution of **(E)** Alu, **(F)** LINE1, and **(G)** LTRs, normalized against the number of such repeats. The bars at the base of the ridge indicate the individual repeat element of that specific size (x-axis).

Differential abundance of repeat elements is one of the major features of the lncRNAs. Moreover, lncRNAs often modulate gene expression through repeat elements present within the lncRNA sequences (8). Therefore, to understand the role of repeat element-based modulation of the lncRNA functions, we looked at the repeat element distribution within the interacting lncRNAs (n=34) discovered from the co-expression network. We compared, i) the class/subclass of these repeat elements, ii) their presence in the sense/antisense strand, and iii) their sizes with the repeat elements present within the co-expressed but non-interacting lncRNAs (n=524) (**Figure 3A**). We observed that the interacting lncRNAs have significantly higher abundance of repeat elements compared to the non-interacting lncRNAs (p value = 0.048) (**Figure 3B**). We observed a significantly higher presence of short interspersed nuclear elements (SINEs) (p value < 0.0001), long interspersed nuclear elements (LINEs) (p value < 0.0001), and the long terminal repeats (LTRs) (p value < 0.0001) in the interacting lncRNAs (**Figure 3C**, **Supplementary Table S7**).

Interestingly, the presence of Alu was significantly higher in the interacting lncRNAs (p value < 0.0001), and within those, majority of the Alus were present on the sense strand. The LINE1 was also significantly higher in the interacting lncRNAs (p value < 0.0001), however, the majority of the LINE1 was present in the antisense strand. Alus are known to modulate immune response by homologous interaction with the immune responsive genes, and to activate interferon response (48, 49). While some studies highlight the accumulation of LINE1 upon viral infection, LINE1 is reported to activate the antiviral response in specific instances (50, 51). Besides, the antisense LINE1 is reported to regulate the expression of surrounding genes (52). The LTRs help in viral replication, and it is also reported to activate host immune response (53, 54). Since the interacting lncRNAs are expressed in the VBT group, the higher abundance of Alu, LINE1, and LTR elements within the interacting lncRNAs suggest a possible regulation of antiviral and immune response by the interacting lncRNAs in the vaccination breakthrough individuals.

Since the Alu element was significantly abundant in the interacting lncRNAs, we looked at the sub-classes of Alu elements. We observed that the Alu S, but not Alu J and Alu Y were significantly abundant (p < 0.0001) in the interacting lncRNAs (**Figure 3D**). Evolutionarily, Alu J is the oldest amongst the Alu family, while Alu Y is the youngest one, and Alu S is of intermediate age. Interestingly, the majority of Alu-mediated function is reported to be mediated by Alu S, therefore, higher abundance of Alu S in the interacting lncRNAs indicate multi-dimensional Alu-mediated gene regulatory functions (55, 56). Since the Alu-mediated gene regulation is exerted relatively better by the full length Alus, we looked at the size distribution of Alus between the interacting and the non-interacting lncRNAs. We defined Alus above 250 bps as full length Alus. Importantly, we observed higher abundance of full length Alus in the interacting lncRNAs (p value = 0.019) (**Figure 3E**). Together, these evidences suggest that the Alu-mediated functions are mediated by full length Alu S in the vaccination breakthrough. Additionally, we also checked the size of the LINE1 and LTRs between the vaccination breakthrough and the unvaccinated and observed a higher abundance of LINE1 up to 1kb, and LTRs up to 500 bps in the interacting lncRNAs, although statistically non-significant (**Figures 3F, G**). Overall, the majority of LINE1 were up to 2kb size, and LTRs up to 1kb, which conforms their global distribution within the Human genome (LINE 1 mean size 0.9 kb, LTRs size range 200-500bps).

### Alu and LINE1-driven homology-based interaction between the lncRNAs and the TAD genes

**Figure 4:**
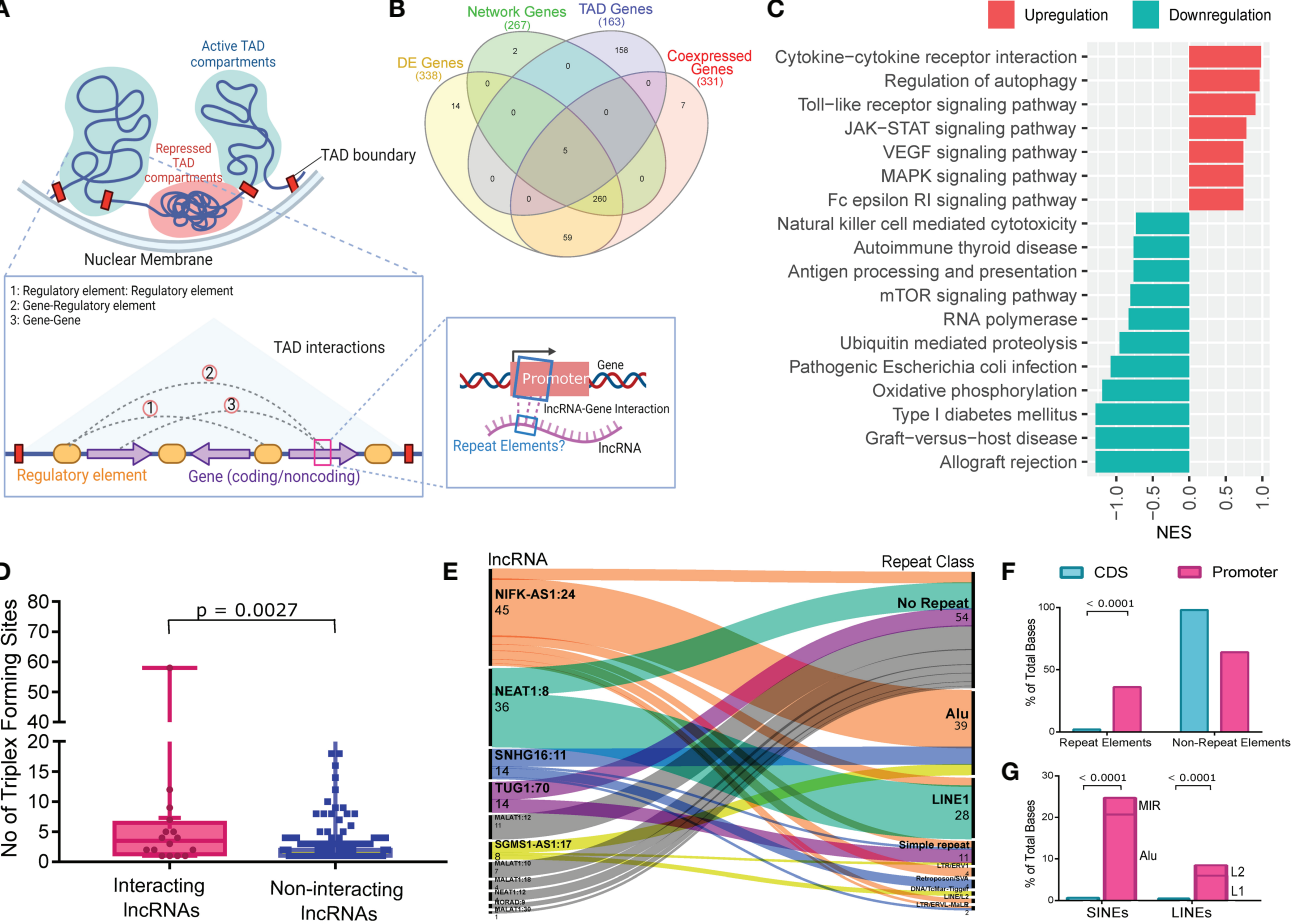
LncRNAs Interaction with the TAD genes and Repeat elements abundance at the Interaction sites. **(A)** Illustration of the TAD, type of interactions within the TAD and homology-based interaction between the lncRNA and promoter region of genes from the same TAD. **(B)** Venn diagram showing the number of protein coding genes from different steps of analysis and their overlap. **(C)** GSEA of the genes from the same TAD as of the interacting lncRNAs. The x-axis represents the Normalized Enrichment Score (NES), while the color represents the expression of the pathways. **(D)** Number of Triplex Forming Sites (TFS) between interacting lncRNAs and non-interacting lncRNAs. Upper bar shows statistical significance. **(E)** Repeat element abundance at the site of interaction within lncRNAs. The number of interacting sites within the lncRNAs and the corresponding repeat elements at these sites are represented in the alluvial plot on left and right sides, respectively. **(F)** Barplot showing the repeat element distribution between the promoter region and CDS region of the interacting TAD genes. Total bases covered by repeat elements were normalized against the total bases of the promoter and CDS. **(G)** Stacked bar plot showing the abundance of SINEs and LINEs; and Alu and LINE1 within SINEs and LINEs respectively, between the promoter and the CDS region. L1 and L2 represent LINE1 and LINE2 respectively. Upper bar shows statistical significance.

Repeat elements are known to have homology-based interaction which can regulate expression of a gene which may be present in a distant genomic loci, but within the 3D proximity (57). It is one of the possible ways in which repeats exert their gene regulatory functions. Genes in 3D proximity inside a TAD may interact with one another, either directly or indirectly *via* regulatory components. Taking these factors into consideration, we examined the lncRNA interaction with the genes present in the same TAD as the lncRNAs, as well as the existence of repeat elements within the interacting regions, to understand the type of interaction/s (**Figure 4A**).

Initially, we looked at the genes present within the TADs of the interacting lncRNAs (n=34) and identified 163 protein coding genes (**Supplementary Table S8**). Of these, we found only five genes overlapping with the DE genes in the vaccination breakthrough patients (**Figure 4B**). Therefore, we performed GSEA (using KEGG database) of the genes present within TADs to understand the possibly perturbed biological processes. Interestingly, we observed immune and inflammatory response associated pathways (such as NK cell mediated cytotoxicity, Antigen processing and presentation, mTOR signaling) to be suppressed (**Figure 4C**, **Supplementary Table S9**).

We then checked for the association between the interacting lncRNAs and the genes from the same TADs. We used Triplex domain finder from Regulatory Genomics Toolbox to find triplex forming sites (TFS) between the lncRNAs and the promoter region of the genes, since interaction at the promoter region regulates gene expression and thereby modulating gene function (29). We compared the number of lncRNA-Gene TFS between the interacting lncRNAs and the non-interacting lncRNAs. The interacting lncRNAs were found to have significantly higher (p value = 0.0027) numbers of TFS with the promoter region of genes from the same TADs (**Figure 4D**). Out of the 34 interacting lncRNAs, we found 11 lncRNAs to have interaction with the 19 TAD genes at the promoter region. The interaction sites within the 11 lncRNAs were subsequently characterized to determine the repeat elements distribution throughout the interacting sites. Interestingly, Alu and LINE1 were the most prevalent repeat elements in the interacting regions of the lncRNAs as well as no repeat elements were found at some of the interaction sites (**Figure 4E**, **Supplementary Table S9**).

Finally, we looked at the repeat element abundance at the promoter region of the interacting TAD genes and compared it with the repeat element abundance within the CDS region of the genes. The promoter region was found to have significantly higher abundance of repeats compared to the CDS of the same genes (**Figure 4F**, **Supplementary Table S10**). Out of the total repeat elements, Alu and LINE1 were found to be significantly enriched in the promoter region of the genes as compared to the CoDing Sequences (CDS) (**Figure 4G**). Taken together, these findings suggest an Alu and LINE1 driven homology-based interaction between the lncRNA and the promoter region of genes from the same TAD as an additional possible mechanism of the lncRNA-mediated immune response regulation.

## Discussion

**Figure 5:**
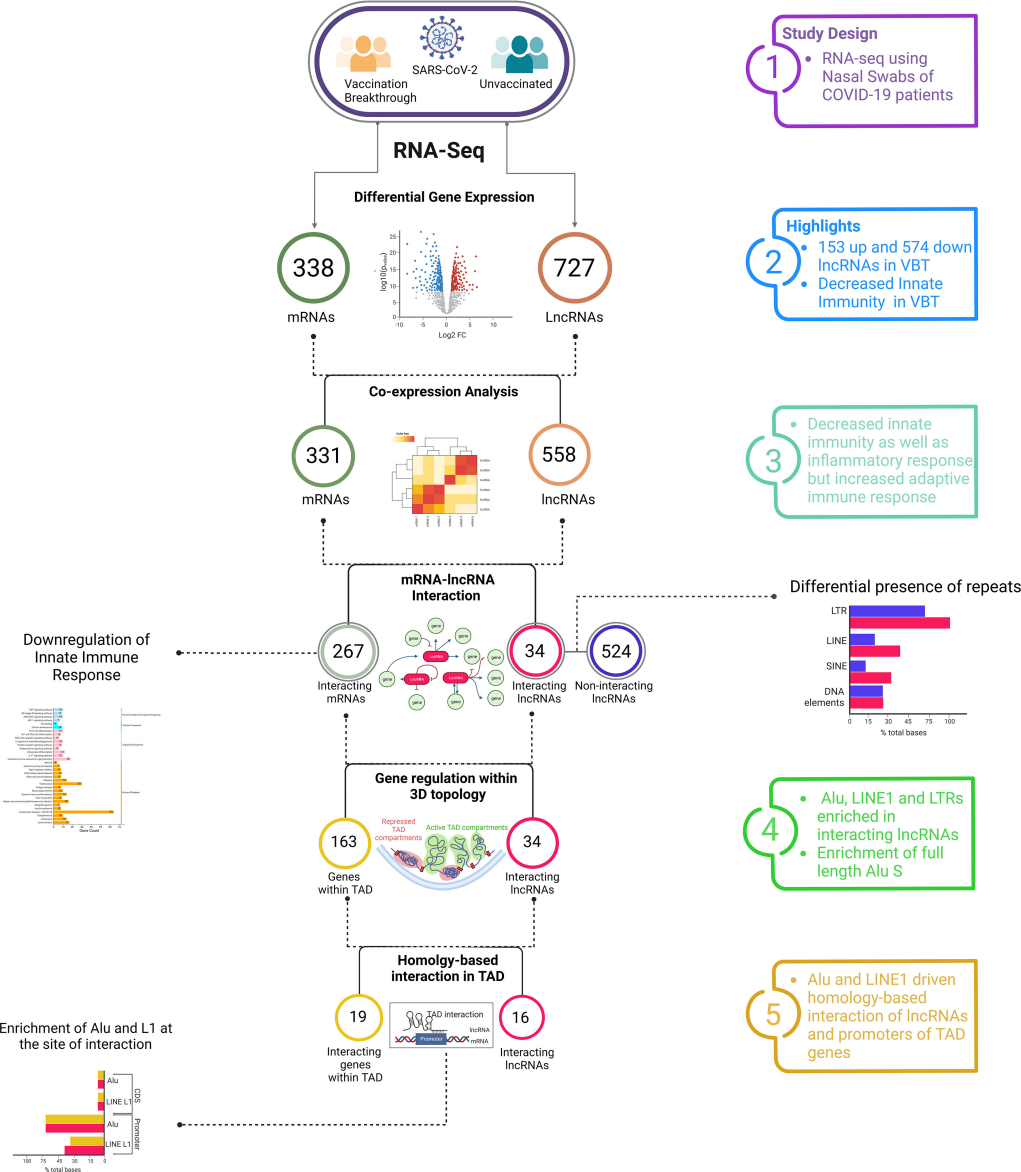
Summary of the findings threading together the hierarchical inferences for vaccination breakthrough SARS-CoV-2 infections. Through a series of inter-connected and combinatorial analysis, the findings highlight the functional role of lncRNAs in the VBT, albeit with milder disease severity.

With the emergence of multiple VOCs and their differential ability to evade the immune system, the cases of SARS-CoV-2 vaccination breakthrough infection is increasing rapidly. The antibody and cytokine-mediated immune and inflammatory response to infection are often considered as a single layered response, however, in reality, the host response is a more complex, multi-layered response, mediated by a cascade of transcriptomic, proteomic and cellular alterations. Though multiple literatures demonstrated the antibody-mediated immune response dynamics in vaccination breakthrough infection, little is known about the transcriptomic regulation of immune system, especially the non-coding RNA-mediated immune regulation. In this study, using transcriptomic analysis of the unvaccinated and the breakthrough SARS-CoV-2 infections, we attempted to understand the differential host response in the breakthrough infection. Our study has been able to highlight the lncRNA-mediated differential immune and inflammatory response in the breakthrough infection, which might explain the vaccination breakthrough. Through our DE analysis of the lncRNAs, mRNAs and lncRNA-mRNA co-expression analysis, we found an increased adaptive immune response, but decreased innate immune and inflammatory response in the VBT. Repeat element abundance within the DE lncRNAs and repeat element-mediated homologous interaction between the lncRNAs and genes from same TADs further support the findings, as well as highlight a possible way of lncRNA-mediated modulation of gene expression (**Figure 5**).

The clinical data of the vaccination breakthrough patients highlight that despite having higher viral load, they have milder symptoms compared to the unvaccinated COVID-19 patients. These indicate that the vaccination does have a protective role in the COVID-19 patients. However, the question: despite receiving the immunizations that were meant to protect them/us from infection, what enables the virus to infect, remains unclear.

**Figure 6:**
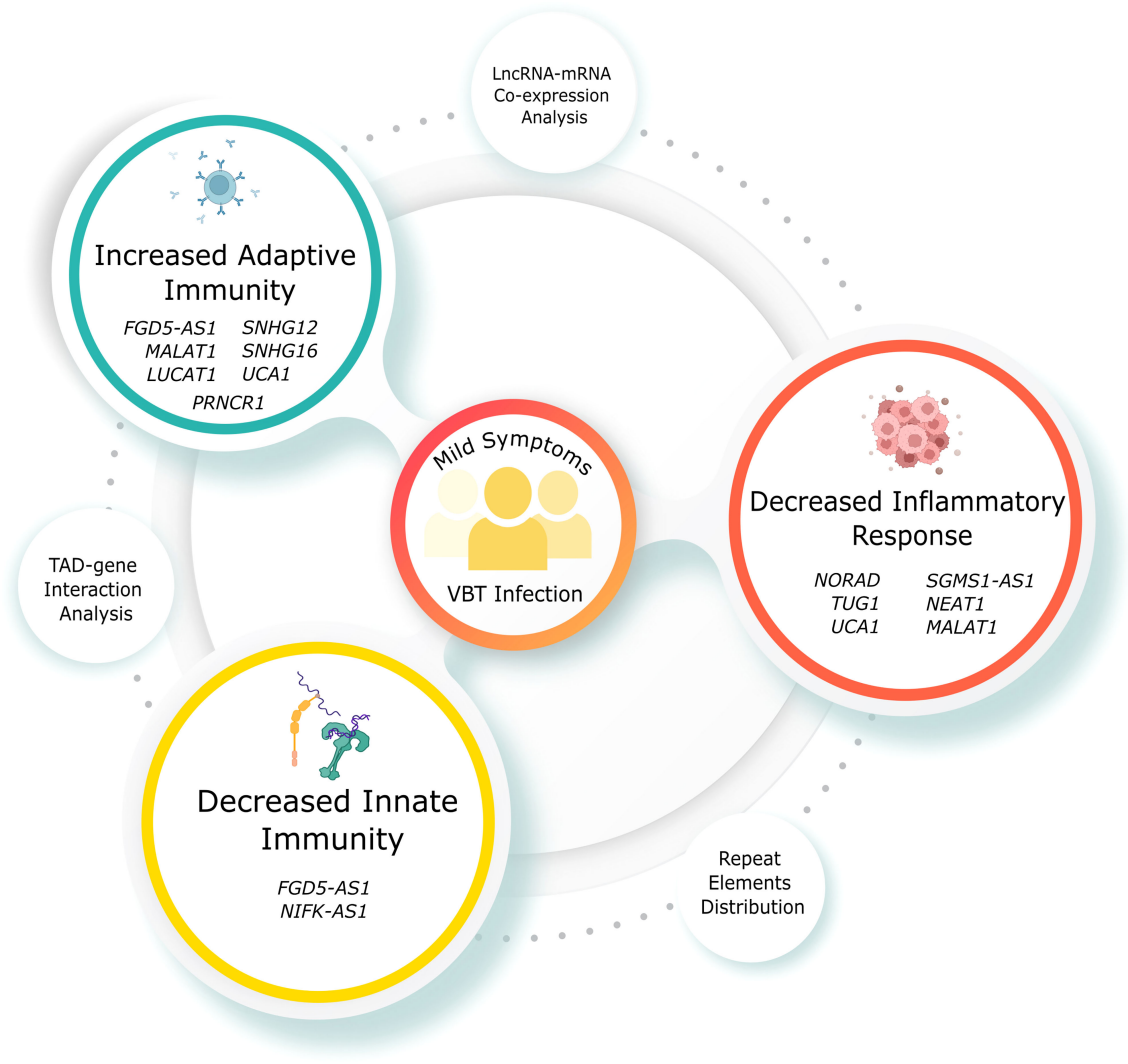
Mechanism explaining the mild symptoms in VBT SARS-CoV-2 infection. Didactic figure showing the specific lncRNAs modulating the immune and inflammatory response resulting in the mild symptoms in the VBT group.

**Figure 7:**
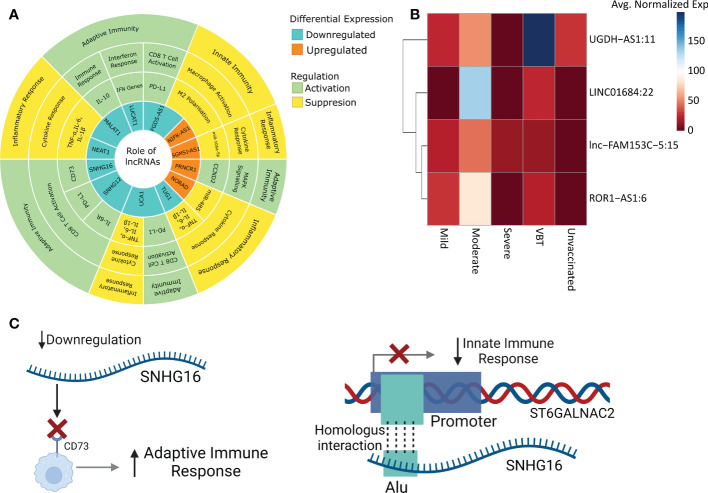
Summary and possible mechanism of the lncRNA functions. **(A)** Summary of the lncRNA functions, represented with respect to the VBT group. **(B)** Average normalized expression of selected lncRNAs across two study cohort. **(C)** Mechanisms of lncRNA-mediated immune modulation, taking *SNHG16* as an example.

Through transcriptomic analysis, we identified 153 upregulated and 574 downregulated lncRNAs in the VBT. While few of the DE lncRNAs are known to regulate immune responses in infected individuals, some lncRNAs are reported to regulate the immune responses in other disease contexts. For instance, lncRNA *PRNCR1*, upregulated in the vaccination breakthrough, induces MAPK signalling, a key signalling in the adaptive immune response (58). Downregulation of *SNHG12* is known to activate CD8 T cell, thereby activating adaptive immune response in the VBT group (43). Downregulation of another SNHG family member, *SNHG16*, is also known to activate adaptive immunity through CD73 (44). On the other side, *FGD5-AS1* and *NIFK-AS1*, both are known to suppress M2 polarization, a key component of innate immunity in the VBT group (41, 59). Apart from the immune response modulation, lncRNAs *NORAD*, *TUG1* and *SGMS1-AS1* are known to suppress the inflammatory response, while *UCA1*, is known to suppress inflammatory response and activate adaptive immune response (60–63). Both *MALAT1* and *NEAT1* might be crucial for regulating the immune system and IL-6 mediated inflammation which is one of the key immune pathways that responds to the SARS-CoV-2 infection (64). Besides, *NEAT1* also regulates the inflammation by modulating inflammasomes such as *NLRP3*, *NLRC4*, and *AIM2*, which may alter the immunological response to the COVID-19 infection (65). *LUCAT1*, apart from regulating the IFN genes, also regulates NF-kB dependent genes by modulating the JAK-STAT pathway. Another lncRNA, *PIRAT*, regulates the expression of alarmins *S100A8/A9* in the monocytes, which are essential for the pathogenesis of COVID-19 (66). Apart from the previously discussed regulation of immune and inflammatory response, *MALAT1* is also known to regulate the IFNG gene regulation by modulating HIF-1 through has-miR-155-5p (67). It is also interesting to note that *MALAT1* negatively regulates proliferation and migration of endothelial progenitor cells in deep vein thrombosis (DVT) (68). The unusual presence of acute DVT post vaccination could have been associated with the downregulation of *MALAT1* in the vaccinated individuals. Overall, the concerted lncRNA expression seems to be suppressing the innate immune and inflammatory response, while activating the adaptive immune response (**Figures 6**, **7A**). Innate immune response is activated within minutes of antigen exposure, and acts as a first line of defence while adaptive immune response is triggered after a few hours or days of first antigen exposure. Therefore, a suppressed innate immune response in the VBT group might explain the SARS-CoV-2 infection in the vaccinated individual. At the same time, activated adaptive immune response and suppressed inflammatory response possibly explains the milder symptoms in the VBT group. We have compared the expression of study specific lncRNAs between the two reinfection cases (within VBT group) and other VBT infection with no prior infection. Overall, the expression pattern did not change between the two groups, except for few lncRNAs (**Supplementary Figure S2**). For example, the expression of MALAT1:18, MALAT1:12, and MALAT1:28 were different between the two groups. However, expression of 6 other MALAT1 transcripts were similar across the groups. This is possibly because of the differences in number of samples in each group (2 vs 26). Besides, since 6 out of 9 transcripts remained similar, and the fact that the functional interpretation was at gene level, not at individual transcript level, the findings are not influenced by these two samples. Overall, the findings highlight the lncRNA-mediated immune responses as one of the explanations of the milder severity in VBT infection.

Apart from the DE-lncRNAs with known functions with respect to either direct or indirect immune/modulation response modulation, a very large number of lncRNAs still don’t have annotated functions. While the known DE-lncRNAs are suggesting towards a decreased innate immune/inflammatory response and increased adaptive immune response, the pathway enrichment analysis of all the DE-lncRNAs, which essentially utilizes the knowledge-based gene-lncRNA interaction for enrichment, do suggest that the lncRNAs with unknown functions are also relevant to infectious disease etiology. Enrichment of pathways which are not directly associated with the DE-lncRNAs discussed earlier, such as Apoptosis, Hedgehog signalling, VEGF signalling, and Tight junction pathways, and the high number of genes enriched in these pathways suggests a lncRNA-mediated regulation of cellular homeostasis in the infected individuals. Therefore, further understanding of these functionally unannotated lncRNAs holds potential.

In our previous study, we have highlighted lncRNA-mediated immune responses modulating COVID-19 differential disease severities (8). Notably, four lncRNAs, *lnc-FAM153C-5:15, ROR1-AS1:6, LINC01684:22*, and *UGDH-AS1:11* upregulated in the VBT group, were downregulated in the Severe group compared to Mild or Moderate (**Figure 7B**). As discussed, VBT group is milder than the unvaccinated group, and upregulation of these four lncRNAs in the milder group follows a similar expression profile in the Mild/Moderate group compared to the severe group. This also suggests a lncRNA-mediated modulation of disease severity in both the cohorts. Association of two functionally unannotated lncRNAs (*lnc-FAM153C-5:15, LINC01684:22*) with disease severity also reiterates the importance of understanding their function.

Repeat elements are known to be involved in the hierarchical gene regulatory activities. Besides, high repeat element abundance is a feature of the lncRNAs. Our previous study as well as few other groups also highlight the role of repeat elements in the infectious disease and its association with disease severity [8,11,33,34]. Previously, we have also shown that in severe COVID-19, repeat element abundance within the DE lncRNAs are higher than their global abundance within the lncRNAs (8). Here also we observed higher abundance of repeat elements in the interacting lncRNAs, which are involved in the immune-modulation of the VBT patients. Multiple studies reported the involvement of Alu and LINE1 repeats in infectious diseases and in modulating the immune response (49, 50). Interestingly, LTRs are involved in viral replication and act as inhibitors of innate immune response (54). Therefore, the specific abundance of Alu, LINE1 and LTR elements reiterates the downregulation of innate immune responses in the VBT group.

The eukaryotic genome is folded in complex chromatins, which is essentially through topologically associating domains (TAD). Within TAD, genes that are present in a distant genomic loci, are actually present in 3D proximity, and have a higher chance of functionally interacting with each other. Often, repeat elements are the key to this interaction, due to their ability to facilitate homologous recombination with their counterparts present within a different gene. The GSEA of the genes present within the TADs of the interacting lncRNAs highlighted the decreased innate immune and inflammatory response in the VBT patients. Majority of the TAD genes were significantly downregulated in the VBT, however, the log2 fold change was low. When we investigated for the functional importance of the association between interacting lncRNAs and these TAD genes, we found that the interacting lncRNAs interact with the promoter region of its TAD genes, and we observed a higher abundance of Alu and LINE1 at the interaction site, both on the lncRNAs and the promoter region of the genes. Few studies reported downregulation of gene expression by repeat element-mediated homologous interaction (69). This supports our hypothesis that Alu and LINE-driven homologous interaction of lncRNAs and promoter region of TAD genes downregulated the expression of those genes, resulting in activation of adaptive immune response but downregulation of innate immune and inflammatory responses. This also highlights the lncRNA-mediated immune modulation by multiple ways.

For example, *SNHG16*, downregulated in the VBT group, activates adaptive immune responses by CD73. At the same time *SNHG16* interacts with the promoter of *ST6GALNAC2* gene, which is present within the same TAD. Interestingly, both on the interacting region of *SNHG16*, and the promoter region of *ST6GALNAC2*, Alu element was enriched, and *ST6GALNAC2* was downregulated in the VBT group. Notably, *ST6GALNAC2* was downregulated in the VBT group, and *ST6GALNAC2* is involved in innate immune response, thereby suggesting suppression of innate immune response (**Figure 7C**). Overall, we observed a concerted modulation of innate and adaptive immune responses, as well as inflammatory responses by the lncRNAs in the VBT patients, and repeat elements as a key modulator of the lncRNA-mediated immune regulation.

While we highlight the above findings, it is important to highlight the possible ways of future strengthening of the findings. The study is based on the nasopharyngeal RNA collected from the COVID-19 patients at the day of hospital admission/home quarantine. While this helps understanding the initial host response at the site of entry, availability of blood samples, and samples post-hospitalization could have increased our understanding of the adaptive immune response. Besides, the neutralizing antibodies could not be measured due to unavailability of the blood samples. Analyzing the neutralizing antibody could shed more light on the adaptive immune response mounted by the vaccination. Additionally, availability of a similar clinical cohort could have helped to validate the findings in a different cohort using clinico-genomics based approaches.

## Conclusion

The evidences suggest lncRNA-mediated suppression of the innate immune response by regulation of the macrophage activation, and inflammatory responses by suppression of the cytokine production, while the activation of adaptive immune response in the VBT group was mediated by the CD8 T cells and IFNG gene regulation. The evidences also suggest multiple ways of lncRNAs-mediated immune modulation, including the lncRNA-mRNA co-expression, repeat element distribution and repeat element mediated homology-based interaction with the TAD genes. Overall, the lncRNA-mediated immune and inflammatory response modulation might provide another explanation of the breakthrough and milder symptoms in the vaccinated COVID-19 patients in addition to the antibody-mediated immune modulation.

## Data availability statement

The data presented in the study are deposited in the NCBI SRA repository https://www.ncbi.nlm.nih.gov/, accession number PRJNA868733.

## Ethics statement

The studies involving human participants were reviewed and approved by the Institutional Ethics Committee of Both CSIR-Institute of Genomics and Integrative Biology, and Max Super Speciality Hospital, under the approval number CSIR-IGIB/IHEC/2020-21/01. The patients/participants provided their written informed consent to participate in this study.

## Author contributions

PC: Investigation, Formal analysis, Writing - Original Draft, Visualization. PaM: Formal analysis, Visualization. PrM: Formal analysis, Visualization. JS: Data Curation, RG: Formal analysis, BT: Resources, SB: Resources, RP: Conceptualization, Writing - Review & Editing, Supervision, Funding acquisition. All authors contributed to the article and approved the submitted version.

## Funding

The study was funded by two grants from the Bill and Melinda Gates Foundation (INV-033578 and INV-030592).

## Acknowledgments

The authors duly acknowledge all the COVID-19 patients who participated in the study. Authors acknowledge the help and support from Dr. Aradhita Baral towards facilitation as research manager and coordination with the funders. Authors acknowledge the support of Anil Kumar and Nisha Rawat towards COVID-19 sample transport and sample management. Partha Chattopadhyay acknowledged the CSIR for their research fellowship.

## Conflict of interest

The authors declare that the research was conducted in the absence of any commercial or financial relationships that could be construed as a potential conflict of interest.

## Publisher’s note

All claims expressed in this article are solely those of the authors and do not necessarily represent those of their affiliated organizations, or those of the publisher, the editors and the reviewers. Any product that may be evaluated in this article, or claim that may be made by its manufacturer, is not guaranteed or endorsed by the publisher.

## Supplementary material

Supplementary Table S1 Clinical and demographic details of the study participants.

Supplementary Table S2 Differential expression of lncRNAs between VBT and unvaccinated.

Supplementary Table S3 Differential expression of mRNAs between VBT and unvaccinated.

Supplementary Table S4 Pearson correlation analysis of DE lncRNAs and mRNAs.

Supplementary Table S5 GSEA of network interacting genes.

Supplementary Table S6 Pathway enrichment of network interacting lncRNAs.

Supplementary Table S7 Repeat element distribution across interacting and non-interacting lncRNAs.

Supplementary Table S8 LncRNAs and corresponding TAD genes.

Supplementary Table S9 LncRNA-TAD gene interaction at promoter region.

ASupplementary Table S10 Repeat element distribution between CDS and promoter region of TAD genes.
