# Supplementary figures and images for: Transcriptomic study reveals lncRNA-mediated downregulation of innate immune and inflammatory response in the SARS-CoV-2 vaccination breakthrough infections
Source: Front Immunol. 2022 Nov 18;13:1035111. doi: 10.3389/fimmu.2022.1035111 (PMC9716354; doi:10.3389/fimmu.2022.1035111)

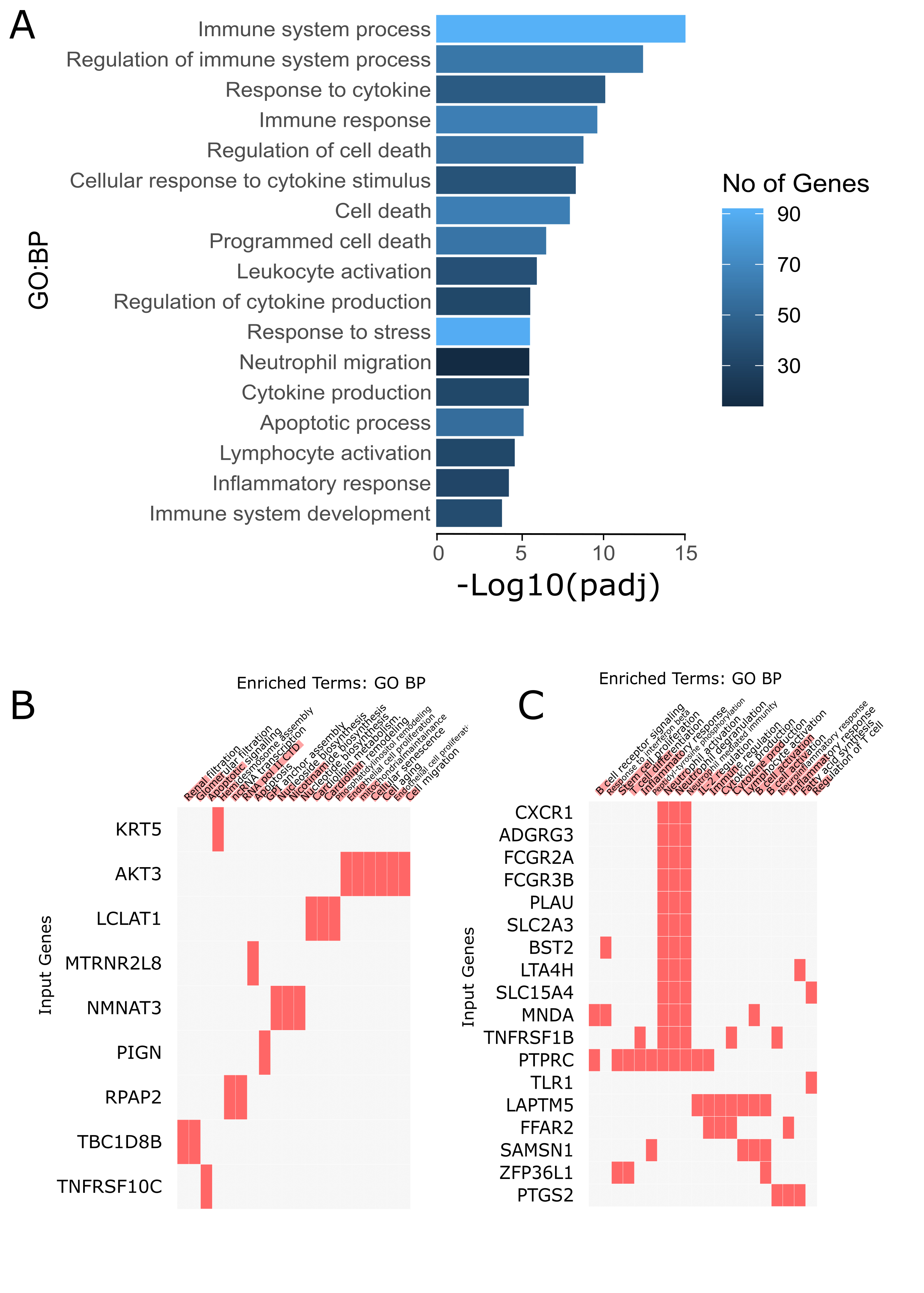

Supplement: Supplementary file 1 [file Image_1.jpeg]

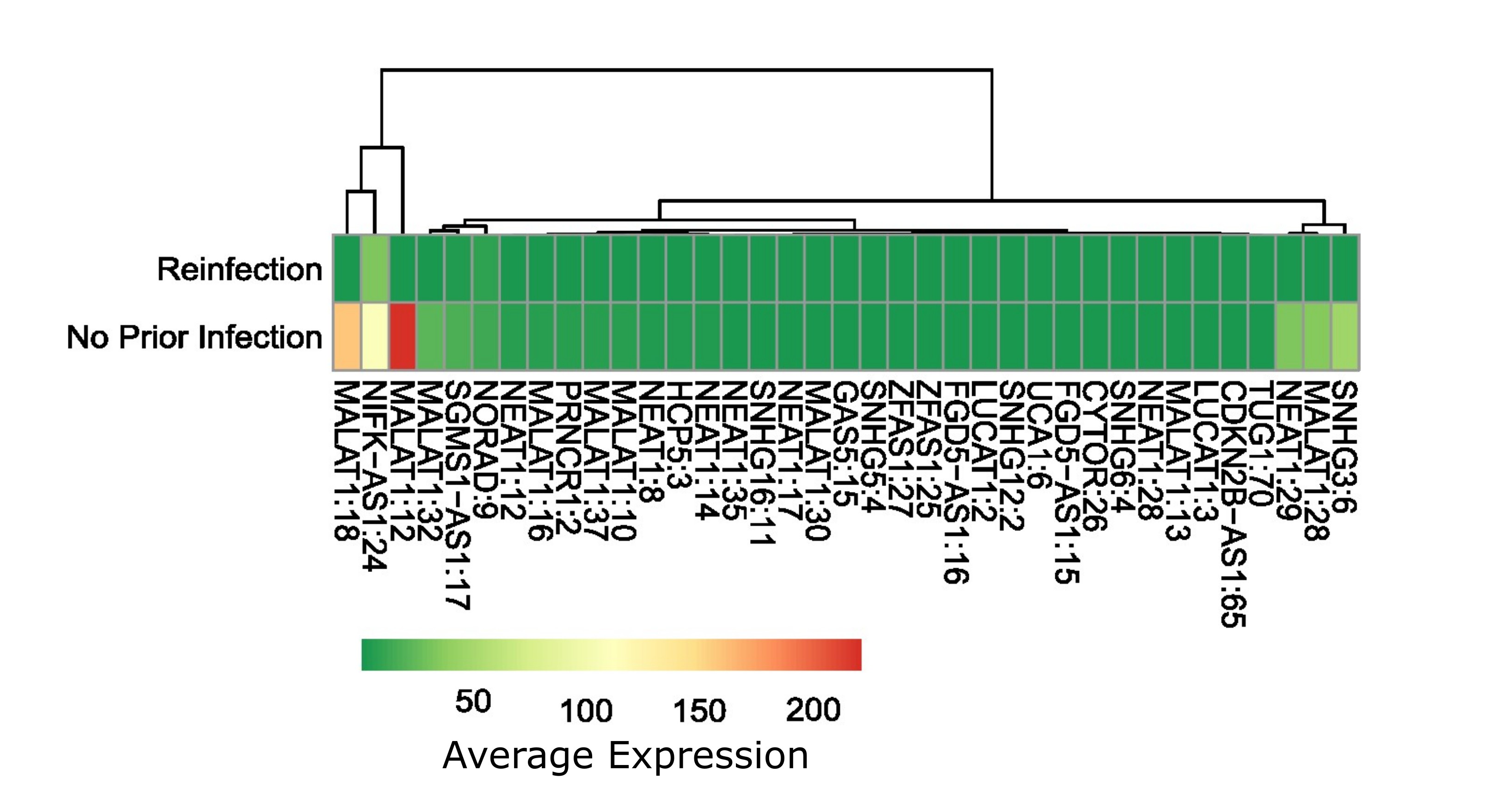

Supplement: Supplementary file 2 [file Image_2.jpeg]
